# Clinical trials in cancer screening, prevention and early diagnosis (SPED): a systematic mapping review

**DOI:** 10.1186/s12885-023-11300-8

**Published:** 2023-09-04

**Authors:** Emma L. O’Dowd, Samuel W. D. Merriel, Vinton W. T. Cheng, Sam Khan, Lynne M. Howells, Dipesh P. Gopal, Elizabeth A. Roundhill, Paul M. Brennan, Philip A. J. Crosbie, Richard D. Neal, Karen Brown, Emma J. Crosbie, David R. Baldwin

**Affiliations:** 1 Department of Respiratory Medicine, Nottingham University Hospitals NHS Trust, Hucknall Road, Nottingham, NG5 1PB UK; 2 Centre for Primary Care & Health Services Research, University of Manchester, Oxford Road, Manchester, M13 9PL UK; 3 Leeds Institute of Medical Research, University of Leeds, Leeds, UK; 4 Leicester Cancer Research Centre, University of Leicester, Leicester, LE2 7LX UK; 5 Primary Care Unit, Centre for Primary Care, Wolfson Institute of Population Health, Queen Mary University, London, UK; 6 Children’s Cancer Research Group, Leeds Institute of Medical Research, University of Leeds, Leeds, UK; 7 Translational Neurosurgery, Centre for Clinical Brain Sciences, University of Edinburgh, Edinburgh, UK; 8 Division of Infection, Immunity & Respiratory Medicine, University of Manchester, Manchester, UK; 9 Thoracic Oncology Centre, Manchester University NHS Foundation Trust, Manchester, UK; 10 Exeter Medical School, St Luke’s Campus, University of Exeter, 1.12 College House, Magdalen Road, EX1 2LU Exeter, UK; 11 Department of Obstetrics and Gynaecology, St Mary’s Hospital, Manchester University NHS Foundation Trust, Oxford Road, Manchester, M13 9WL UK; 12 Division of Cancer Sciences, Faculty of Biology, Medicine and Health, The University of Manchester, Oxford Road, Manchester, M13 9WL UK

**Keywords:** Cancer, Screening, Prevention, Early detection of cancer, Trials, Systematic mapping

## Abstract

**Background:**

Global annual cancer incidence is forecast to rise to 27.5 M by 2040, a 62% increase from 2018. For most cancers, prevention and early detection are the most effective ways of reducing mortality. This study maps trials in cancer screening, prevention, and early diagnosis (SPED) to identify areas of unmet need and highlight research priorities.

**Methods:**

A systematic mapping review was conducted to evaluate all clinical trials focused on cancer SPED, irrespective of tumour type. The National Cancer Research Institute (NCRI) portfolio, EMBASE, PubMed and Medline were searched for relevant papers published between 01/01/2007 and 01/04/2020. References were exported into Covidence software and double-screened. Data were extracted and mapped according to tumour site, geographical location, and intervention type.

**Results:**

One hundred seventeen thousand seven hundred one abstracts were screened, 5157 full texts reviewed, and 2888 studies included. 1184 (52%) trials focussed on screening, 554 (24%) prevention, 442 (20%) early diagnosis, and 85 (4%) a combination. Colorectal, breast, and cervical cancer comprised 61% of all studies compared with 6.4% in lung and 1.8% in liver cancer. The latter two are responsible for 26.3% of global cancer deaths compared with 19.3% for the former three. Number of studies varied markedly according to geographical location; 88% were based in North America, Europe, or Asia.

**Conclusions:**

This study shows clear disparities in the volume of research conducted across different tumour types and according to geographical location. These findings will help drive future research effort so that resources can be directed towards major challenges in cancer SPED.

**Supplementary Information:**

The online version contains supplementary material available at 10.1186/s12885-023-11300-8.

## Introduction

Cancer is the second leading cause of mortality worldwide, accounting for approximately one in six deaths. In 2018 there were 9.6 million cancer related deaths [1]. Cancer incidence and mortality are rising worldwide due to a combination of factors. The ageing and growing population, accompanied by a relative decline in mortality from stroke and cardiovascular disease are major contributors but there are also changes in the prevalence of certain risk factors, many of which are related to behavioural and socioeconomic influences [2]. Global annual cancer incidence is forecast to rise to 27.5 M by 2040, a 62% rise from 2018 [3]. Prevention interventions will help attenuate the rise in incidence and improved early detection can reduce cancer-related mortality.

There is a strong scientific rationale that underpins cancer screening, prevention, and early diagnosis (SPED) research but globally it is under-resourced compared with research funding for new treatments [4]. Whilst considerable progress is being made in systemic therapy for cancer, prevention and early detection are the most effective ways to reduce mortality for most cancers. A study using data from the United Kingdom found that 38% of cancers had a potentially preventable cause [5]. The effectiveness of prevention is illustrated by the huge reduction in mortality from tobacco-related cancers in countries where smoking rates are declining [6]. The challenge is to find ways in which existing prevention strategies can be improved and other modifiable risk factors can be identified and addressed. In early diagnosis, the aim is to detect and treat cancer before it has metastasised, when cure is most likely. Pre-malignant and minimally invasive lesions contain fewer genetic alterations compared to advanced disease and exhibit less tumour heterogeneity [7]. Later diagnosis may therefore be expected to confer a high mortality due to these factors that drive both growth, inherent resistance to treatment, and tendency to metastasise. In addition, the rising costs of treatment for cancer makes prevention and earlier diagnosis important from a health economic perspective.

There is thus a clear rationale for pursuing SPED interventions as a powerful way of reducing global cancer mortality. To ensure equity in outcomes across all cancer types, it is paramount to focus national research and funding agendas to address areas of unmet need [8]. Generating an understanding of the current research portfolio in this area will aid governments, healthcare policy-makers, research funding bodies, and cancer-related charities achieve this goal. The aim of this systematic mapping review was to:

1. Identify all clinical trials in cancer SPED;
2. map these into cancer type and geographic location;
3. ascertain future research priorities by identifying disparities between number of trials and cancer incidence, mortality, and geographic location.

These priorities were identified by members of the NCRI SPED advisory group, which includes clinicians, researchers, and public and patient representatives.

## Methods

**Fig. 1:**
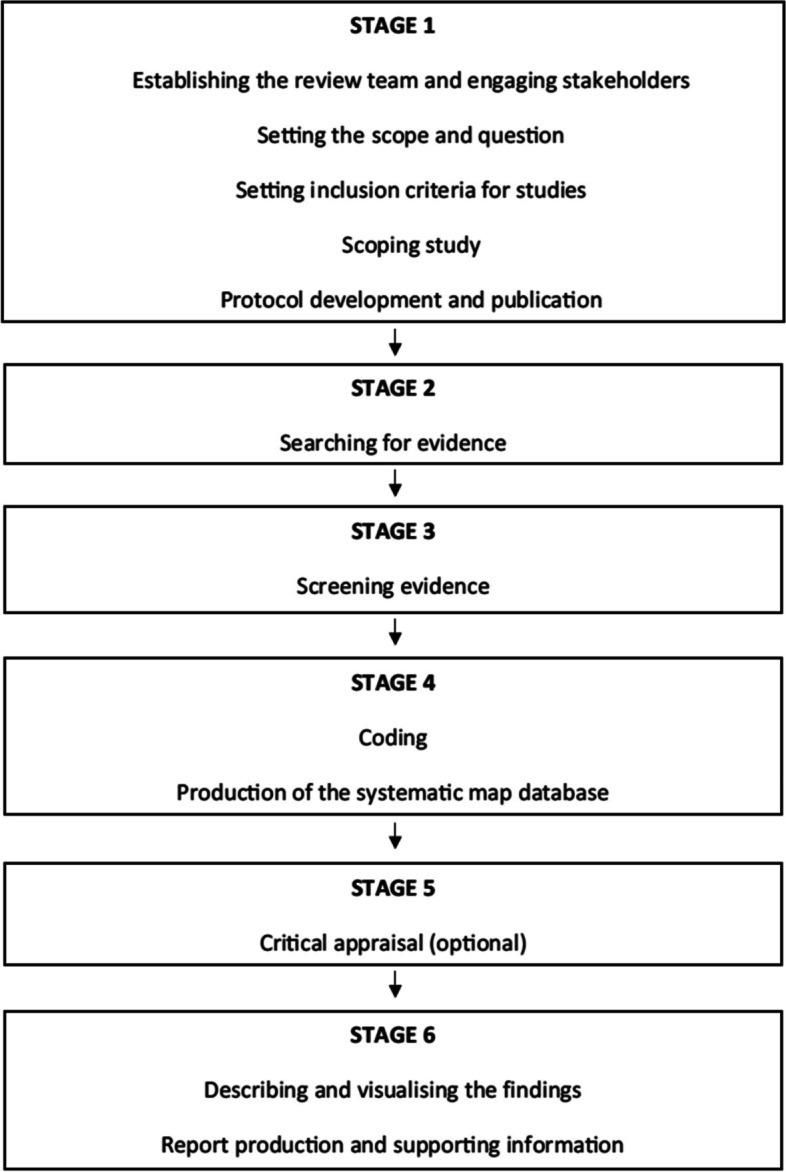
Stages of a systematic mapping review [10]

A systematic mapping review approach was followed to provide a comprehensive overview of published trials in cancer SPED and to allow research gaps and under-researched populations to be identified. Systematic mapping reviews ‘categorise existing literature on a particular topic and identify gaps in research from which to commission further reviews and/or primary research’ [9]. The stages followed were closely aligned to the process outlined by James et al. (see Fig. 1) [10]. Given the scale of the research aims and likely heterogeneity in trial design, aims and outcome measures, a systematic mapping review was also deemed most appropriate to summarise the existing published studies in this field.

### Search strategy

The following databases were searched for relevant studies published between 01/01/2007 and 01/04/2020: NCRI portfolio, EMBASE, PubMed and Medline. The search strategy was tested in a scoping review of PubMed, then developed iteratively using key terms related to cancer screening, prevention, and early diagnosis to maximise the number of relevant articles retrieved without missing any (see Supplementary information). Hand-searching of relevant reference lists was performed to ensure complete capture, and first authors were contacted for further information on included studies where necessary. Members of NCRI Clinical Studies Groups (CSGs) also signposted authors to key studies within their relevant cancer areas to ensure key studies in cancer SPED were captured.

All search hits were imported into Covidence (https://www.covidence.org) for screening and data extraction.

### Selection criteria

Clinical trials, including pilots and feasibility studies, assessing an intervention focused on cancer SPED irrespective of tumour type were eligible for inclusion in this review. Trial interventions could involve one or more cancer types and could be targeted at an individual or population level. Observational studies nested within trials of cancer SPED were also eligible and identified as a distinct category. Table 1 shows the definitions that were employed for screening search hits for inclusion.

**Table 1:** Definitions of cancer screening, prevention, and early diagnosis

Screening	Performing a test or tests for cancer detection in individuals without symptoms of the disease to achieve an early-stage diagnosis
Prevention	Primary – reduction in one or more cancer risk behaviours in individuals without a diagnosis of cancer; therapeutic prevention in high-risk groups (for example those with genetic predisposition) Secondary – treatment and/or monitoring of pre-malignant conditions that might develop into cancer
Early diagnosis	Interventions aimed at achieving an earlier diagnosis of cancer, regardless of whether the individual has symptoms of the disease or not

Observational studies not performed using a clinical trial cohort were excluded, as were case series, conference abstracts, study protocols, commentaries, letters, or correspondence. Cancer treatment trials, studies of non-malignant tumours and studies published or registered in any language other than English were also excluded.

### Screening

Titles and abstracts of search hits from each database were independently assessed by two reviewers from the study team (VC, LH, SK, DG, ER, SWDM, EOD) against the inclusion/exclusion criteria. Full text papers were acquired and reviewed independently by two reviewers. Any discrepancies at title and abstract, or full text stage were resolved by a third reviewer.

### Data extraction and analysis

Data extraction and coding from full text articles of included studies into Covidence was performed using pre-specified data fields, including study year, aims, design, number of subjects in each arm, country(s), population, sample size, intervention type (SPED), tumour(s), and funding source (if declared). Extracted data were then categorised and mapped according to tumour site, country of study, and intervention type. Critical appraisal was not performed, as is the norm with systematic mapping reviews [10].

### Role of funding source

There was no funding source for this study. Access to Covidence was funded by the National Cancer Research Institute.

## Results

### Literature search and study selection

**Fig. 2:**
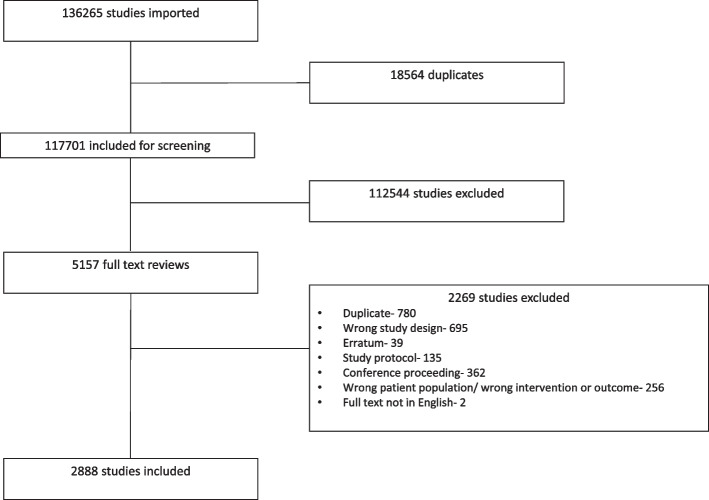
PRISMA flow chart

The electronic search strategy identified 136,265 potential studies for inclusion. After removing duplicate studies, 117,701 abstracts were screened and 112,544 were excluded as they did not meet the pre-specified inclusion criteria. Amongst the 5,157 full-text articles assessed for eligibility, 2,888 were included for analysis. Figure 2 shows the PRISMA flow chart summarising this process.

### Summary of the studies selected

Of the studies included, 2,265 were trials of screening, prevention, early diagnosis, or a combination of these (hereafter referred to as mixed) and 623 were nested studies within trials, described separately.

### Trials (excluding nested studies)

Randomised controlled trials comprised 77% of the total, with non-randomised trials making up the remainder. Publications by year were evenly spread between 2007 and 2019, with the largest number being 220 (10% of the total) in 2019 and the lowest being 128 (6%) in 2008. Table 2 shows the breakdown of published trials within each of the 4 categories.

**Table 2:** Proportion of studies by category

Category	Number	Proportion
Screening	1184	52%
Prevention	554	24%
Early diagnosis	442	20%
Mixed	85	4%

**Fig. 3:**
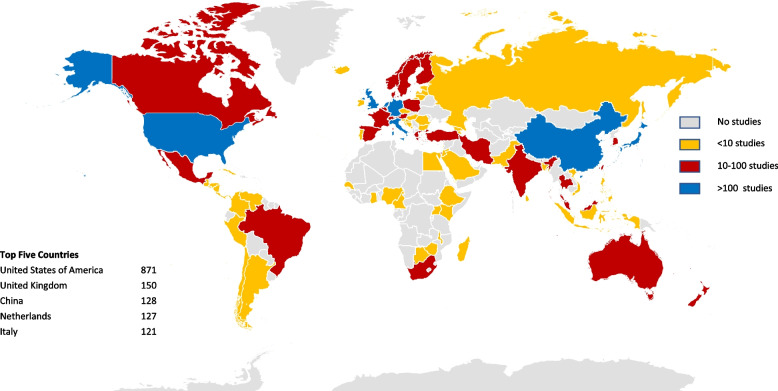
Density map of geographical spread of trials

**Fig. 4:**
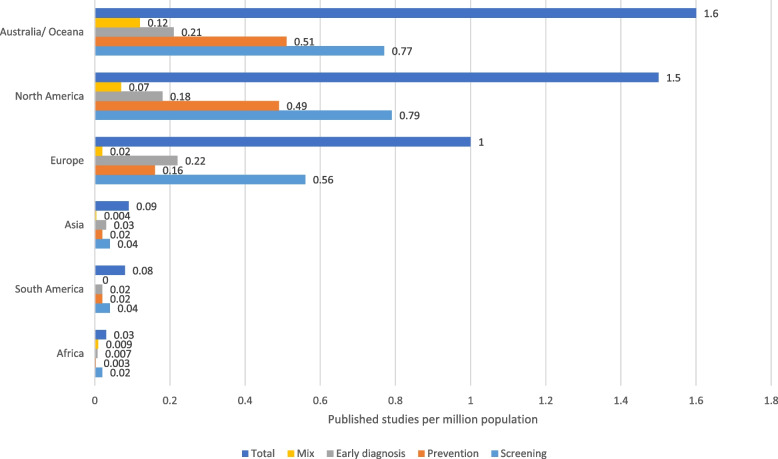
Published trials overall and by category per million population

The geographical distribution of trials showed that 40% of trials were based in North America, 32% in Europe and 16% in Asia. These data are summarised as a density map by country in Fig. 3. To account for differences in population density between countries, the number of trials per million population is presented in Fig. 4. The most common tumour types by country were also reviewed. In North America, Europe, Australasia, and Asia, colorectal was the most common cancer type studied, accounting for between 32 and 36% of all published studies. In the Middle East, breast cancer was the most common (33%), whereas in South America and Africa cervical cancer studies dominated, accounting for 53% and 71% of all published trials respectively. In fact, in Africa, cervical cancer and breast cancer accounted for 88% of all studies. For all countries (apart from multi-country studies which comprised only 3% of the total) screening research was most common, ranging from 46% in Asia to 62% in Africa. Prevention research accounted for 50% of the multi-country studies compared with only 10% of African studies. Early diagnosis trials were least common in North America (12%) and most common in Asia (34%).

**Fig. 5:**
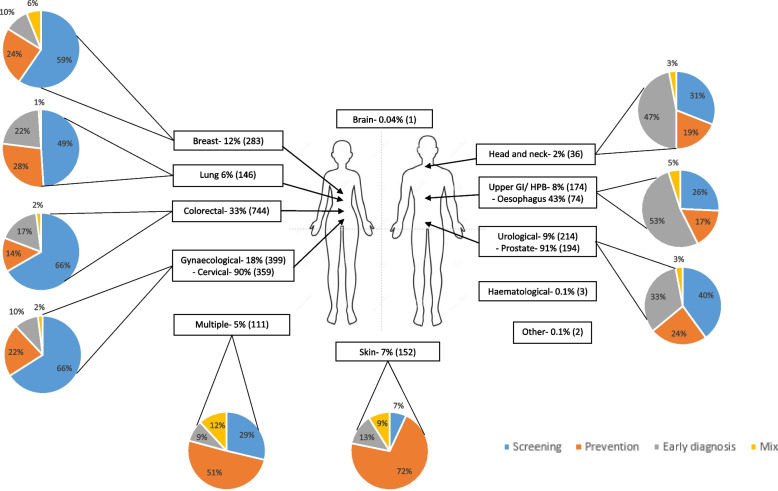
Trial breakdown by tumour site and stratification by category. The pie charts show the distribution for each tumour type by category of screening, prevention, early diagnosis, or mixed

More detailed data were collated for each tumour site. The number and overall proportion of trials per tumour type stratified by category is provided in Fig. 5. Due to small numbers for brain tumours (1 study), haematological (3 studies) and other cancer types (2 studies), which comprised 1 insulinoma and 1 neuroendocrine malignancy, a more in-depth breakdown was not performed. Multiple tumours that are managed by the same sub-speciality team or originate from the same anatomical area were grouped together in the same category (Table 3). For all tumour sites the top 3 interventions investigated by the trials were health promotion (25%), pharmacological interventions (16%) and endoscopic procedures (14%). Total number of trial participants ranged from 3 to 1,555,000. A breakdown of these data by tumour type is summarised in Table 3.

**Table 3:** Summary of trial characteristics by tumour site

Tumour Type																		
	**Colorectal**		**Upper gastrointestinal/ hepatobiliary**		**Urology**		**Breast**		**Gynaecological**		**Head and neck**		**Lung**		**Multiple**		**Skin**	
	Number (*N* = 744)	%	Number (*N* = 174)	%	Number (*N* = 214)	%	Number (*N* = 283)	%	Number (*N* = 399)	%	Number (*N* = 36)	%	Number (*N* = 146)	%	Number (*N* = 111)	%	Number (*N* = 152)	%
**Intervention**	Endoscopy (242)	33	Endoscopy (53)	30	Pharmacological (50)	23	Health promotion (107)	38	Diagnostic test (88)	22	Imaging (10)	28	Imaging (62)	42	Health promotion (50)	45	Health promotion (63)	41
	Health promotion (206)	28	Imaging (40)	23	Imaging (42)	20	Imaging (74)	26	Pharmacological (79)	20	Diagnostic test (9)	25	Health promotion (24)	16	Pharmacological (28)	25	Pharmacological (35)	23
	Pharmacological (83) Behaviour change (50) Imaging (43) Molecular test (29) Biomarkers (15) Questionnaire (12) Standard of care (9)	11 7 6 4 2 2 1	Diagnostic test (27) Pharmacological (20) Health promotion (14) Biomarker (10) Surgery (5)	16 12 8 6 3	Diagnostic test (32) Health promotion (29) Biomarker (14) Standard of care (11) Molecular test (11) Behaviour change (8) Prediction tool (6)	15 14 7 5 5 4 3	Pharmacological (41) Behaviour change (30) Lifestyle (8) Diagnostic test (7) Standard of care (4)	14 11 3 2 1	Health promotion (79) Molecular test (53) Standard of care (24) Imaging (12) Biomarker (11) Endoscopy (10) Questionnaire (8) Prediction tool (5)	20 13 6 3 3 3 2 1	Pharmacological (6) Biomarker (4) Endoscopy (2) Behaviour change (2)	17 11 6 6	Pharmacological (17) Behaviour change (10) Biomarker (9) Endoscopy (8) Diagnostic test (7) Questionnaire (4) Risk prediction (2)	12 7 6 5 5 3 1	Behaviour change (8) Lifestyle (6) Questionnaire (4) Imaging (4) Diagnostic test (3) Risk prediction (2)	75 4 4 3 2	Behaviour change (24) Diagnostic test (9) Imaging (7) Questionnaire (7) Light therapy (3)	16 6 5 5 2
**Geographical area (top 3)**	North America (298)	40	Asia (70)	40	Europe (87)	41	North America (138)	49	Europe (127)	32	Asia (17)	47	Europe (62)	43	North America (66)	59	North America (87)	57
	Europe (260)	35	Europe (45)	26	North America (86)	40	Europe (70)	25	North America (117)	29	Europe (9)	25	North America (58)	40	Europe (22)	20	Europe (34)	22
	Asia (125)	17	North America (43)	25	Asia (25)	12	Asia (26)	9	Asia (62)	16	North America (8)	22	Asia (20)	14	Asia (12)	11	Australasia (20)	13
Subtypes within each category (where relevant)*			Biliary (1)	1	Bladder (16)	7			Cervical (359)	90	Head and neck NOS (3)	8						
			Gastric (35)	20	Prostate (194)	91			Endometrial (19)	5	Laryngeal (1)	3						
			Liver (40)	23	Renal (2)	1			Ovarian (19)	5	Nasopharyngeal (6)	17						
			Oesophagus (74)	42	Testicular (2)	1			Vaginal (2)	0.5	Oral (20)	56						
			Pancreas (24)	14					Vulval (1)	0.3	Thyroid (6)	17						
**Total participants**	**Range**		**Range**		**Range**		**Range**		**Range**		**Range**		**Range**		**Range**		**Range**	
	3- 362,165		18- 184,786		14- 721,299		11- 1,198,493		15- 860,028		12- 183,000		20- 154,901		8- 1,555,000		4- 360,288	

*Total percentage may be greater than 100% due to rounding. NOS- not otherwise specified

**Fig. 6:**
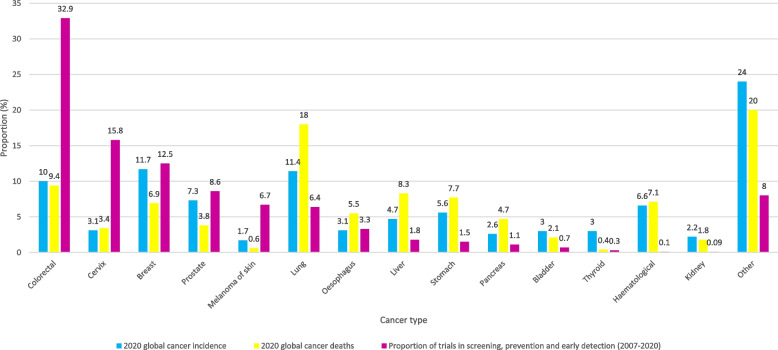
Global cancer incidence, mortality (from Globocan for 2020) [11] and proportion of screening, prevention and early detection trials by tumour type

To identify tumour subgroups that are underrepresented in trials when considering their incidence and mortality rates, global cancer data for 2020 were obtained from The Global Cancer Observatory [11]. Figure 6 shows a comparison of the global cancer incidence, mortality and proportion of trials in screening, prevention, and early detection by tumour type. Results from nested studies and trial funding are summarised in the Supplementary material.

## Discussion

This study mapped all trials published between January 2007 and April 2020 investigating SPED for cancer. Cancer types with established screening programmes had the greatest number of trials, with colorectal, breast and cervical cancer comprising 61% of all published research. In fact, a third of all published trials identified in this study were colorectal cancer-specific, whilst some tumour types are hardly represented, if at all (for example brain tumours with only 1 published trial and sarcoma with no identified trials). Two common cancers, lung and prostate cancer, made up a further 15% of all trials, both with a considerable number of screening trials. When 2020 global annual cancer incidence and mortality rates were compared to number of published trials, there were striking differences: cervical cancer screening and prevention trials (15.8%) were fivefold higher than its relative incidence (3.1%) and mortality (3.4%), while colorectal trials were threefold higher for both incidence and mortality. By comparison, only 6.4% of research activity involved lung cancer and 1.8% liver cancer, tumours that account for 18% and 8.3% of cancer deaths respectively. There are also inequities in trial geographic location, with 88% of the studies based in North America, Europe, or Asia compared with only 2% in Africa and 1.5% in South America. However, when trials are considered per million population Australasia, North America, and Europe are substantially better represented than Asia, South America, and Africa, suggesting that more work needs to be done to increase funding opportunities and address research infrastructure in these areas. Furthermore, in developing countries in Africa, South America, and the Middle East, research on cervical cancer and breast cancer dominate with few published trials in any other tumour type. It may be that this high number of trials in these two tumour sites is appropriate because mortality from breast and cervical cancer is much higher in Africa than in developed countries. However, our findings highlight the need to address research in other tumour types in these countries [2].

Most tumour types have a much higher proportion of screening research compared to prevention or early diagnosis trials; for example, 66% of the trials for colorectal and gynaecological cancers were screening trials. This reflects the success of these screening programmes. For skin cancer, prevention research dominates (72%) and for head and neck and upper gastrointestinal/ hepatobiliary cancers early diagnosis trials were relatively more common (comprising 47% and 53% of the total respectively). Interventions are mostly focused on health promotion, testing pharmacological agents, endoscopic procedures, or imaging (68%). There needs to be a greater focus on prevention research, particularly since obesity rates are climbing globally and smoking prevalence remains high in some countries and is rising in others. However, for some tumour types, prevention research is a challenge because the aetiology is poorly understood and stratifying individuals according to risk is challenging. Furthermore, there is a lack of surrogate biomarkers of cancer that can be used as endpoints in intervention studies.

In addition to identifying areas of unmet need, this study highlights duplication of effort in some areas, with very similar trials taking place in different settings. For example, there were 242 studies looking at endoscopy in colorectal cancer, which accounts for 11% of all published trials in cancer SPED. Further research may not be needed because results from one trial are transferrable to other countries allowing funders of research to prioritise filling research gaps and stimulating more collaborative approaches. In some settings national studies are required to provide evidence for local implementation but there should be more effort to ensure that study outcomes are transferrable to different healthcare systems.

### Comparison with existing literature

Begum et al. [12] performed a bibliometric analysis of cancer research papers published between 2002 and 2013 to quantify research activity in the 28 European Union Member States and Iceland, Norway, and Switzerland (EUR31). They showed that the amount of research activity was strongly correlated with gross domestic product (GDP), with the leading funding sources being government (30%), followed by private non-profit organisations (19%). Like this study, they showed that lung, oesophageal and pancreatic cancers were under-represented in research, particularly given their incidence and poor outcomes. Only 1.7% of lung cancer research papers identified in this study focused on screening, compared with 8% of breast cancer papers.

A further bibliometric study focussed on research outputs from 10 countries in the Middle East and North African (MENA) region and showed an increase in cancer research outputs between 2011 and 2018, although the authors did not analyse breakdown by tumour type or type of research [13]. Aggarwall et al. used bibliometrics to look at lung cancer research outputs between 2004 and 2013 for 24 countries [14]. During this time frame lung cancer research outputs increased year on year and accounted for 5.6% of all cancer research in 2013, similar to the 6.4% shown in this study. Screening and diagnosis research contributed a small proportion of the reported studies, with most focused-on genetics, biomarkers, and treatments. Again, the authors highlighted the disparity between cancer incidence, mortality, and proportion of research spending on lung cancer.

Funding bodies recognise that some cancers with poor outcomes have not received a proportionate amount of funding for screening, prevention, and early diagnosis research relative to their incidence and mortality. For example, Cancer Research UK (CRUK) identified lung, pancreatic, brain and oesophagus as their cancers of unmet need. Although funding allocated to these cancer types has increased as a result of their prioritisation, breast cancer still receives the lion’s share of research spending according to the latest CRUK annual report (15%), followed by lung cancer (13%) and colorectal cancer (12%) [15] In the US, data on call cancer research from the National Cancer Institute from 2018 show approximately 12% of research spend on breast, 7.3% on lung and 5.3% on colorectal cancer [16].

### Strengths & limitations

To our knowledge this is the only study that has mapped the global spectrum of published SPED trials for all cancers. It provides important information about the gaps in funding for some key cancer types, global inequities in research trial activity and a detailed breakdown of the type of research conducted for each tumour type. Due to the size of the study, we were unable to include all published research and had to limit the inclusion criteria to trials (randomised or non-randomised) and studies nested within trials that were also published in English. This means that some areas of SPED research that is better suited to qualitative or non-trial methodology, and those not published in English have not been included. Furthermore, in line with the systematic mapping methodology and the heterogeneous nature of the publications, a critical assessment of the quality of the publications was not performed and we acknowledge that some trials may not be high quality or of sufficient size to produce clinically relevant results.

## Conclusions and future research directions

This study provides a comprehensive overview of the published trials covering cancer SPED over a 14-year period. It shows that there are clear disparities in research effort between tumour types, especially when compared to their relative incidence and mortality rates. The differences imply a considerable discrepancy in funding according to clinical need and the future prioritisation of those cancers with poorest outcomes is important to address health inequalities. The study also shows how few trials have been conducted in some countries and populations, especially developing countries, where other population health interventions may take priority. As we emerge from the SARS-Cov-2 pandemic, late-stage cancer diagnoses are rising because of reduced access to routine screening and diagnostic services and the funds available for research are scarce. Our finding that some areas have received prioritisation (which may be reflected in marked improvements in outcomes), suggests the need for a revised prioritisation exercise. Understanding what research priorities are by identifying under resourced tumour types and gaps in research portfolios is key to reducing inequities and improving outcomes, particularly in those cancer types with increasing incidence and high mortality. This study provides the first global summary that can be used by funders, policy makers and researchers to guide and prioritise future work to try to address both research and funding gaps, with the ultimate aim of reducing the number of cancer deaths.

### Supplementary Information

**Additional file 1.**

## Data Availability

The datasets used and/or analysed during the current study are available from the corresponding author on reasonable request. The search strategy employed is provided in the supplementary appendix.
