# Supplementary material for: Clinical trials in cancer screening, prevention and early diagnosis (SPED): a systematic mapping review
Source: BMC Cancer. 2023 Sep 4;23:820. doi: 10.1186/s12885-023-11300-8 (PMC10476302; doi:10.1186/s12885-023-11300-8)
Supplement: Supplementary file 1 — Additional file 1. [file 12885_2023_11300_MOESM1_ESM.docx]

Table S1: Search strategy

| Database | Search strategy |
| --- | --- |
| Medline via PubMed | (Cancer or neoplasms or tumor or malignancy or carcinoma) and (“Early Detection of Cancer” or prevent* or screen*.mp or detect* or diagnos* or cancer vaccines.mp or biomarkers.mp or early diagnosis.mp) and (Randomized Controlled Trial or Non-Randomized Controlled Trial or Controlled Clinical trial or placebo.mp or Clinical Trial or randomly.mp or trial.mp)  1 January 2007 to 1 April 2020  English Language only |
| EMBASE | (cancer.mp or malignant neoplasm or tumour.mp or neoplasm or tumor.mp or carcinoma.mp or malignancy) and (screening.mp or detection.mp or prevention.mp or awareness.mp or cancer vaccine.mp or biomarker.mp or early.mp or cancer detection.mp or cancer diagnosis) and (crossover procedure or double-blind procedure or randomized controlled trial or single blind procedure or controlled clinical trial.mp or clinical trials.mp)  1 January 2007 to 1 April 2020  English Language only |

Trial funding sources and nested studies

Funding sources for the trials, where reported, were reviewed. In 28% of papers these data were not available. Overall, 38% of trials were government funded, 9% were funded by non-governmental organisations (NGO), 7% industry or charity-funded, 6% academic funded and 3% had no specific funding source. When reviewed by country, government funding was the main source in all but the Middle East or multi-country studies, accounting for 57% of the funding in North America, compared with only 19% in Africa and 2% in the Middle East. Forty-four per cent of multi-country studies were funded by industry. Charitable funding ranged from 17% in Africa to 0% in the Middle East. Overall, most government funding was directed towards colorectal cancer (31%) and breast cancer (13%), whereas industry funding focused more on gynaecological cancer (34%) and colorectal cancer (24%). There was a difference comparing developed and developing countries. For example, government funding in South America was largely directed towards gynaecological cancers, whereas in North America colorectal cancer made up 32% of government-funded SPED research.

### Nested studies within trials

Nested studies within trials were identified separately. An overview of key features of these studies is provided in Table 5. The top 3 interventions tested in these nested studies were biomarkers (18%), pharmacological agents (18%) and imaging (14%).

Table S2: Summary of nested studies within screening, prevention and early detection trials

|  | **Number**  **N= 623** | **Proportion**  **%** |
| --- | --- | --- |
| **Category** |  |  |
| Screening | 354 | 57% |
| Prevention | 198 | 32% |
| Early diagnosis | 48 | 8% |
| Mixed | 23 | 4% |
| **Cancer type (top 5)** |  |  |
| Urology | 140 | 22% |
| Colorectal | 121 | 19% |
| Lung | 117 | 19% |
| Gynaecological | 105 | 17% |
| Breast | 61 | 10% |
| **Trials with studies nested within (top 6)** |  |  |
| Prostate, Lung, Colorectal and Ovarian (PLCO) Cancer Screening Trial | 98 | 16% |
| The National Lung Screening Trial (NLST) | 37 | 6% |
| The European Randomized Study of Screening for Prostate Cancer (ERSPC) | 32 | 5% |
| Prostate Cancer Prevention Trial (PCPT) | 27 | 4% |
| Dutch-Belgian Lung Cancer Screening trial (NELSON) | 24 | 4% |
| Alpha-Tocopherol, Beta-Carotene Cancer Prevention (ATBC) Study | 18 | 3% |
| **Geographic area (top 5)** |  |  |
| North America | 331 | 53% |
| Europe | 221 | 35% |
| Multi-country | 27 | 4% |
| Asia | 20 | 3% |
| South America | 10 | 2% |
